# Membrane Properties Involved in Calcium-Stimulated Microparticle Release from the Plasma Membranes of S49 Lymphoma Cells

**DOI:** 10.1155/2014/537192

**Published:** 2014-01-21

**Authors:** Lauryl E. Campbell, Jennifer Nelson, Elizabeth Gibbons, Allan M. Judd, John D. Bell

**Affiliations:** Department of Physiology and Developmental Biology, Brigham Young University, Provo, UT 84601, USA

## Abstract

This study answered the question of whether biophysical mechanisms for microparticle shedding discovered in platelets and erythrocytes also apply to nucleated cells: cytoskeletal disruption, potassium efflux, transbilayer phospholipid migration, and membrane disordering. The calcium ionophore, ionomycin, disrupted the actin cytoskeleton of S49 lymphoma cells and produced rapid release of microparticles. This release was significantly inhibited by interventions that impaired calcium-activated potassium current. Microparticle release was also greatly reduced in a lymphocyte cell line deficient in the expression of scramblase, the enzyme responsible for calcium-stimulated dismantling of the normal phospholipid transbilayer asymmetry. Rescue of the scrambling function at high ionophore concentration also resulted in enhanced particle shedding. The effect of membrane physical properties was addressed by varying the experimental temperature (32–42°C). A significant positive trend in the rate of microparticle release as a function of temperature was observed. Fluorescence experiments with trimethylammonium diphenylhexatriene and Patman revealed significant decrease in the level of apparent membrane order along that temperature range. These results demonstrated that biophysical mechanisms involved in microparticle release from platelets and erythrocytes apply also to lymphocytes.

## 1. Introduction

Microparticles are small vesicular structures (0.1–1 *μ*m diameter) produced and released by exocytic blebbing of the cell plasma membrane from a variety of cell types including platelets, erythrocytes, leucocytes, endothelial cells, fibroblasts, epithelial cells, and tumor cells [1–4]. Microparticles are detectable basally in the blood of healthy individuals [5], and additional amounts may be shed from cells as a result of activation signals and/or during apoptosis [3, 6]. Microparticles appear to function as mediators of intercellular communication. For example, they may cause cellular activation or apoptosis depending on the target [7–10]. In addition, they are involved in regulation of inflammation, coagulation, and antigen presentation [3, 4, 6]. Hence, they may play a role in the pathogenesis of autoimmune diseases and inflammatory disorders. Moreover, elevated microparticle levels are typically seen in the blood of patients in a variety of disease states such as various cardiovascular disorders including atherosclerosis, diabetes, certain infectious diseases such as HIV, Ebola, and cerebral malaria, and in several cancers [2, 3, 6, 9, 11, 12].

Although small microparticles (less than 100 nm) appear to have an endosomal origin, the majority are larger (100–1000 nm) and are shed through the process of “reverse budding” [13]. In this latter case, release is initiated by a sustained rise in intracellular calcium [1–3, 6]. The mechanism of calcium-stimulated microparticle release has been explored most extensively in platelets and erythrocytes where release requires reorganization of the cytoskeleton, translocation of phosphatidylserine (PS) and other phospholipids to the outer face of the cell membrane, and enhanced permeability to potassium with associated osmotic effects [14–28]. In addition, recent work with erythrocytes has demonstrated that vesicle shedding also depends on physical characteristic of the cell membrane, which can be detected with fluorescent membrane probes sensitive to phospholipid order and organization in the bilayer [29].

The extent to which these various mechanisms apply to nucleated cells has not yet been adequately addressed [3]. Some evidence exists to suggest that the cytoskeletal changes are required in all cells that release microparticles [24–27], and it is reasonable to assume that cytoskeletal attachments would have to be broken for pieces of the membrane to be shed. Whether the other mechanisms (exposure of PS, transmembrane potassium flux, and favorable biophysical properties) are also necessary remains unknown. This study was designed to address that deficiency using S49 lymphoma cells as an experimental model.

## 2. Materials and Methods 

### 2.1. Reagents

Ionomycin, 1-(trimethylammoniumphenyl)-6-phenyl-1,3,5-hexatriene p-toluenesulfonate (TMA-DPH), Alexa Fluor 488 Phalloidin Conjugate, and 6-hexadecanoyl-2-(((2-(trimethylammonium)ethyl)methyl)amino)naphthalene chloride (patman) were obtained from Life Technologies (Grand Island, NY, USA). Ionomycin and MC540 were dissolved in dimethyl sulfoxide (DMSO) as stock solutions, while TMA-DPH was suspended in dimethylformamide. Quinine was purchased from Sigma (St. Louis, MO, USA).

### 2.2. Cell Preparation

S49 mouse lymphoma cells were cultured in DMEM (10% horse serum) at 37°C in humidified air (10% CO_2_). Raji human Burkitt's lymphoma cells were grown at 5% CO_2_ in RPMI (10% fetal bovine serum and L-glutamine). Prior to experiments, unless otherwise stated, cells were isolated through centrifugation then washed and suspended in MBSS (134 mM NaCl, 6.2 mM KCl, 1.6 mM CaCl_2_, 18.0 mM Hepes, 13.6 mM glucose, and pH 7.4 at 37°C) at a density of 0.4–3.0 × 10^6^ cells/mL. Unless stated otherwise, experiments were conducted at 37°C.

### 2.3. Fluorescence Spectroscopy and Light Scatter

Washed cell samples (2 mL) were equilibrated 5 min in quartz fluorometer sample cells in either a Fluoromax 3 (Horiba, Edison, NJ, USA) or PC-1 (ISS, Champaign, IL, USA, anisotropy measurements) spectrofluorometer prior to data acquisition. Sample homogeneity was maintained by magnetic stirring, and temperature was regulated with circulating water baths.

Microparticle release was assayed by light scatter at 500 nm [15]. After data acquisition was initiated, ionomycin (300 nM) was added, and the rate of microparticle release was determined as the slope of the rise in light scatter intensity. That this procedure assesses microparticles released from cells was established previously for S49 cells by differential centrifugation and lipid analysis [30]. Note that typically many of the microparticles released are larger than 500 nm and therefore produce an elevation in the light scatter intensity. However, in rare cases microparticle size is uniformly smaller than 500 nm, and in those instances the shedding of particles produces a negative deflection in the light scatter due to shrinkage of the cells following release.

The fluorescence emission of patman was observed at 435 and 500 nm (250 nM final, excitation = 350 nm) by rapid (3 s resolution) sluing of the emission monochromator mirror. The probe was added to cell samples after measuring background intensity for 100 s, and the fluorescence intensity was then monitored for several hundred seconds until steady state was reached. The polarity of patman's environment was assessed by calculating the generalized polarization (GP) as follows [31]:

(1) $$\begin{array}{c} \text{GP}=\frac{I_{435}-I_{500}}{I_{435}+I_{500}}, \end{array}$$

where *I*_435_ and *I*_500_ are the emission intensities at 435 and 500 nm. The intensity data were smoothed by nonlinear regression to an arbitrary function (sum of two exponentials) prior to calculation of GP.

The steady-state anisotropy of TMA-DPH (250 nM final, excitation = 350, emission = 452) was assessed using Glan-Thompson polarizers. Probe was equilibrated with cell samples for 10 min prior to acquisition of data with excitation and emission polarizers alternatively oriented parallel and then perpendicular to each other. Anisotropy was calculated as described previously, and at least 20 points were averaged in determining values for the figures and statistical analyses [32].

### 2.4. Fluorescence Imaging of Actin Cytoskeleton

Cells were washed and treated with ionomycin as in other experiments. The treated cells were then simultaneously fixed, permeabilized, and stained with fluorescent phalloidin (Alexa Fluor© 488) according to the manufacturer's protocol (Life Technologies). After being mounted onto microscopy slides, cells were stained with a solution containing 165 nM phalloidin, 3.7% formaldehyde, 1% bovine serum albumin, and 0.1 mg/mL lyso-PC (1-palmitoyl-2-hydroxy-sn-glycero-3-phosphocholine) for 20 min at 4°C. Slides were then washed with buffer before coverslips were mounted. Images were collected on an Olympus FluoView FV 300 confocal laser scanning microscope using a 60x oil immersion objective lens. A 3x digital zoom was also applied. The excitation light source was a 488 nm argon laser, and a 505–525 nm bandpass filter was used on the emission detector.

## 3. Results

**Figure 1:**
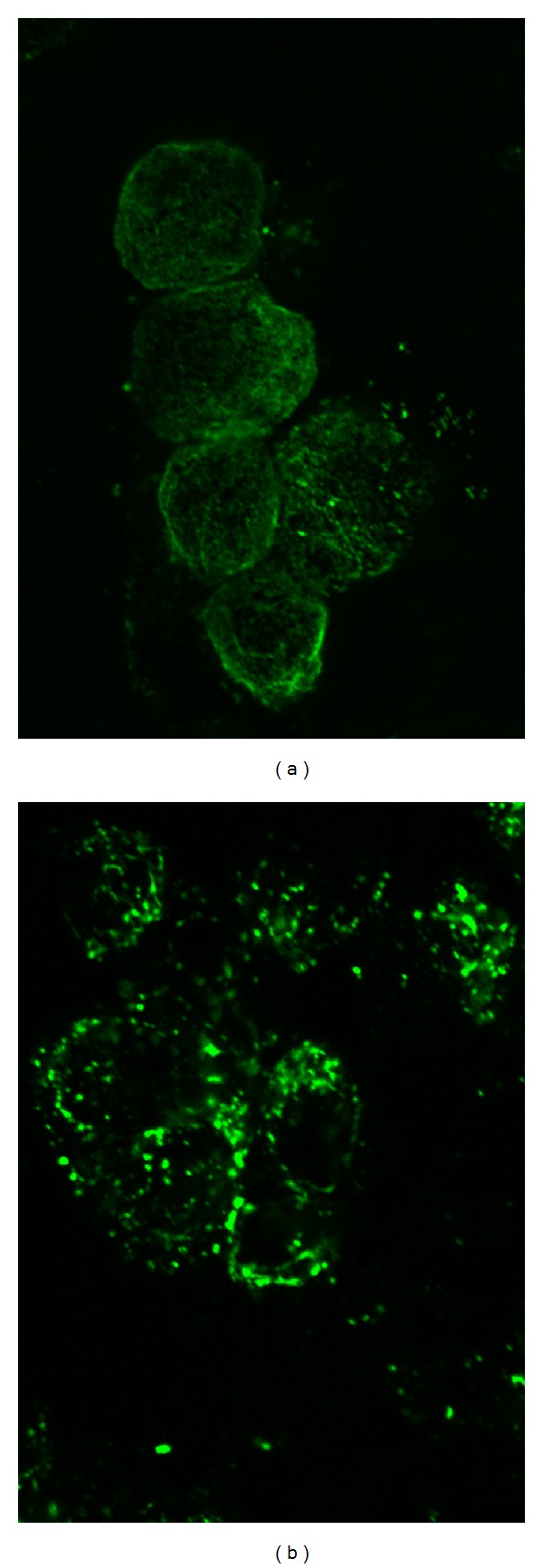
Confocal photographs of actin cytoskeletal without (a) or with (b) ionomycin treatment at 37°C. The actin cytoskeleton of S49 cells was stained with phalloidin.

**Figure 2:**
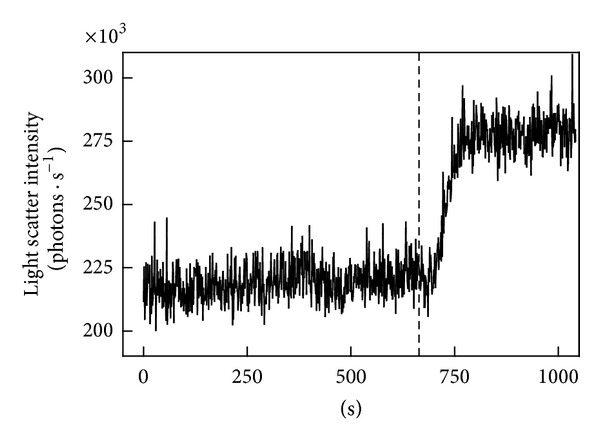
Ionomycin-stimulated microparticle release assayed by light scatter at 37°C. Ionomycin was added at the dotted line. An increase in scatter intensity indicates particle release [30].

**Figure 3:**
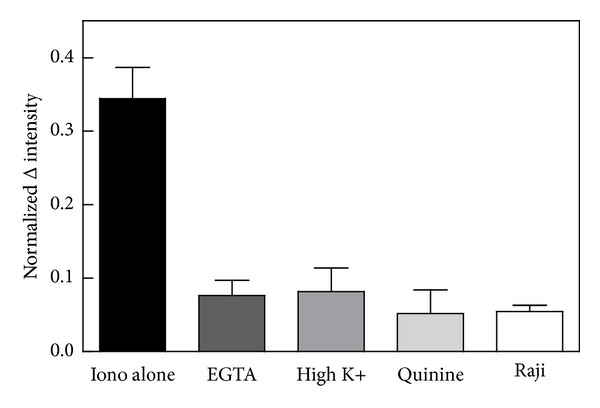
Ionomycin-stimulated microparticle release requires calcium-activated potassium current. Cells were washed and suspended in normal MBSS (“Iono alone” or “Raji”) or in MBSS that contained EGTA (2 mM) instead of calcium, high potassium (83 mM KCl with equivalent reduction in NaCl), or quinine (1 mM) at 37°C. The normalized light scatter intensity was calculated by subtracting the average initial intensity immediately prior to ionomycin addition (20 points) from the average intensity at the plateau after ionomycin (about 350 s later; see Figure 2). This difference was then divided by the average initial intensity to standardize among trials. Differences in the normalized intensity among groups were significant by one-way analysis of variance (*P* = 0.0004, *n* = 2–9 per group). A posttest (Dunnett's) revealed that the group of S49 cells treated with normal MBSS was distinguishable from each of the other four (*P* < 0.05).

Figure 1 displays fluorescence images of the actin cytoskeleton of S49 lymphoma cells before (Figure 1(a)) and after (Figure 1(b)) incubation with a calcium ionophore (ionomycin). As expected based on observations with other cell types [21–27], the cytoskeleton was disrupted rapidly (within 10 min) as indicated by a reduction of extended parallel fibers and an increase of scattered brightly staining actin aggregates. As shown in Figure 2, this disruptive effect of ionomycin was accompanied by a rise in the intensity of light scattered by the sample. This elevation in intensity began within 50 s after addition of ionomycin and continued for another 100 s. Previous work has demonstrated that the light scatter change reflects the release of membrane particles [15, 30].  Figure 3 demonstrates that the difference in light scatter intensity before and after treatment with ionomycin was reproducible and statistically significant (*P* < 0.0001, one-sample *t*-test with *H*_0_ = 0). Moreover, repetition of the experiment in the presence of a calcium chelator (EGTA) inhibited the response to ionomycin and therefore demonstrated that microparticle release was dependent on calcium and therefore not an artifact of the ionophore itself (Figure 3).

### 3.1. Role of Potassium Channels

To determine whether S49 cells require calcium-activated potassium flux for microparticle release similar to platelets and erythrocytes, the experiment of Figure 2 was repeated in the presence of a reduced potassium gradient. In addition, the experiment was repeated with normal potassium concentrations in the presence of the calcium-activated potassium channel blocker quinine [14–16].  Figure 3 shows that both interventions produced about 80% reduction in the amount of microparticles shed from the cells. These results indicated that calcium-activated potassium current was a necessary component of particle release. Presumably, this current is necessary in order to reduce the cell volume osmotically to accommodate the reduced membrane surface area associated with microparticle shedding [33, 34].

### 3.2. Loss of Membrane Lipid Asymmetry

Previous genetic data and experiments with a pharmacological inhibitor of scramblase (R5421, [35]) demonstrated in erythrocytes and platelets that the activity of that enzyme is important for microparticle shedding [19, 29, 36]. Since a scramblase inhibitor is no longer available, the requirement for migration of PS from the inner to the outer leaflet of the cell membrane was tested here for lymphoma cells by using the Raji lymphoma line, which is deficient in scramblase activity [37–40]. The white bar of Figure 3 shows the response to ionomycin in Raji cells. The ionomycin-stimulated change in light scatter intensity in Raji cells was only 16% of that observed in S49 cells. Furthermore, incubation of Raji cells with a higher dose of ionomycin sufficient to restore some of the ability of the cells to translocate PS [40] generated a twofold enhancement of apparent rate of microparticle release (2.1 ± 0.4, *P* = 0.01, *n* = 10). These data argued that PS exposure is also important for microparticle release in nucleated cells.

### 3.3. Membrane Lipid Order

**Figure 4:**
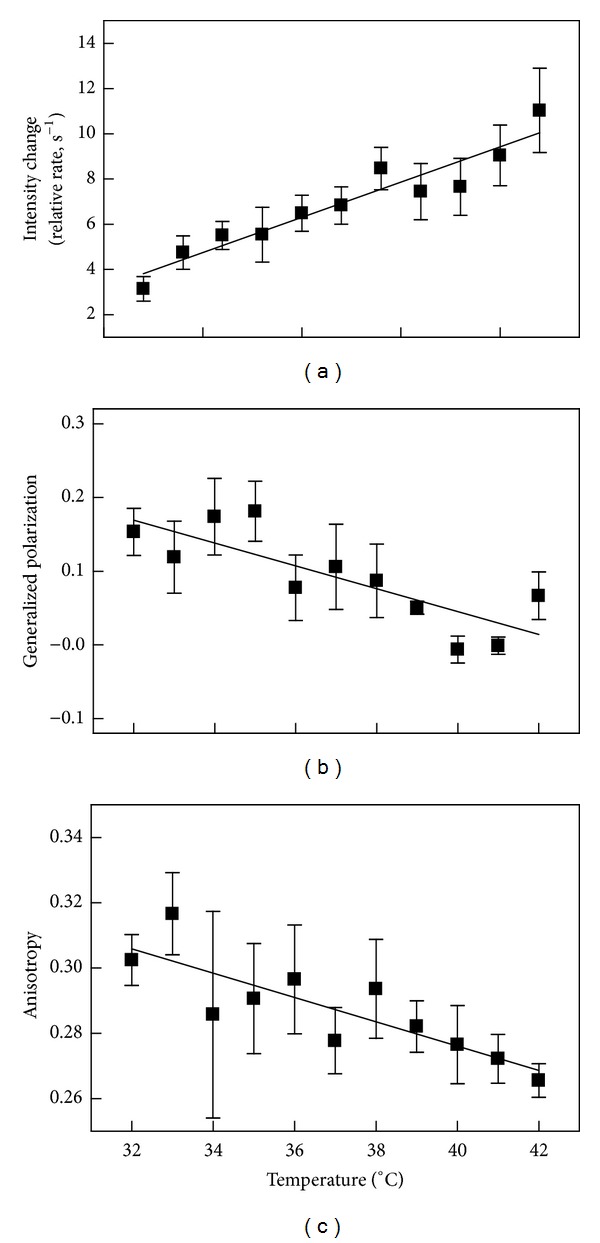
Relationship between the rate of microparticle release and membrane order as a function of temperature. (a) The relative rate of particle release upon addition of ionomycin was calculated from experiments such as that shown in Figure 2. Cells were equilibrated at 37°C and then adjusted to the indicated temperature and equilibrated for 10 min prior to adding ionomycin. The relative rate of release was determined from the maximum slope of the time profile following ionomycin addition and normalized to initial light scatter intensity as in Figure 3. Based on linear regression, the positive trend was significant (*P* < 0.0001, *r* ^2^ = 0.28, *n* = 5–15 per temperature, 83 total values). ((b)-(c)) The experiments of (a) were repeated with cells labeled with patman (b) or TMA-DPH (c). Nonlinear regression to an arbitrary function (sum of two exponentials) was used to smooth the Patman data prior to calculating the value of GP or TMA-DPH anisotropy was averaged from 7 points prior to addition of ionomycin. The negative trends in both cases were significant by linear regression (*P* ≤ 0.006, *r* ^2^ > 0.21, and *n* = 3 per temperature).

Recent studies suggested a role for membrane lipid order and fluidity in determining the ability of erythrocytes to release microvesicles in response to calcium [29]. To explore that possibility for S49 cells, experiments were conducted at various temperatures between 32 and 42°C. As shown in Figure 4(a), the rate of microparticle release increased monotonically with temperature. Linear regression analysis demonstrated that this trend was statistically significant (see legend). Control experiments assessing other known effects of ionomycin (cytoskeletal disruption, phospholipase sensitivity) demonstrated that the drug was equally effective throughout this temperature range (not shown). Therefore, the effects of temperature were presumably on the release process rather than on the efficacy of the drug.

The relative level of membrane order was also assessed at these temperatures by fluorescence spectroscopy using patman GP and TMA-DPH anisotropy. The data with both probes (Figures 4(b) and 4(c)) showed a significant decrease in apparent membrane order. Accordingly, a strong correlation between the level of order detected by these probes and the rate of vesicle release was observed (*P* < 0.025, *r* ^2^ > 0.44, *n* = 11 temperatures, by linear regression, values apply to both patman GP and TMA-DPH anisotropy). Overall, Figure 4 supports the hypothesis that membrane physical properties play a role in microparticle release for both nucleated and nonnucleated cells.

## 4. Discussion

It appears that the basic mechanisms governing microparticle shedding in platelets and erythrocytes may apply broadly to all cell types. This finding implies that microparticle release may simply be the consequence of a reduction in the stability of the cell membrane. Hence, factors that regulate the release process are biophysical in nature and represent withdrawal of elements that normally maintain stability. Based on this study and those focused on platelets and erythrocytes, these critical elements include the cellular osmotic balance, cytoskeletal attachments, the asymmetric distribution of phospholipids across the two faces of the membrane, and the elastic properties of the bilayer [14–27, 29, 41, 42].

The importance of osmotic balance is obvious because of associated effects on cell volume. A cell volume reduction would be required to compensate for the loss of plasma membrane surface area as microparticles are released. Moreover, the volume change would presumably promote membrane budding because of the stress imposed by surface area mismatch. In the case of calcium-induced particle release, the volume reduction appears to involve potassium efflux.

As shown in Figure 1, alterations to the actin cytoskeleton accompanied calcium influx as expected [21–27]. Attempts to verify the importance of this event through pharmacological inhibition of the cytoskeletal alterations (e.g., calpain inhibition) were unsuccessful, probably because of redundant mechanisms for the effect of calcium [3]. In erythrocytes and platelets, where regulatory mechanisms are simpler, inhibition of calpain is sufficient to impair microparticle release [15, 22, 23, 28]. To the extent that these findings apply broadly across cell types [24–27], they imply that one important role of the membrane-associated cytoskeleton is to maintain stability and prevent particle release.

The role of transbilayer asymmetry of phospholipid species in maintaining membrane stability seems less obvious. Loss of that asymmetry during internal calcium accumulation results in exposure of PS on the outer surface. Much attention has been paid to that exposure because of the roles of PS as a signal mechanism in hemostasis and recognition of apoptotic cells by macrophages [43]. Why external exposure of that lipid would be permissive for vesicle release is unclear. In fact, it may be that the critical issue is actually the reduction of PS on the interior of the membrane. Since some proteins with C2-like domains are involved with cytoskeleton-membrane anchoring [44], the reduction of PS on the intracellular membrane face may lead to a loss of critical protein interactions resulting in a diminution of membrane stability. This explanation could rationalize why movement of such a minor component would have such a large impact on the membrane. Moreover, it is possible that PS is not the critical or sole participant in permitting microparticle release since calcium loading will also result in external exposure of phosphatidylethanolamine and intracellular accumulation of phosphatidylcholine through inhibition of aminophospholipid translocase and activation of scramblase [3].

The role of membrane order differed from that of potassium current, cytoskeleton breakdown, and scramblase activation. Each of the latter three appeared required, though not individually sufficient, for microparticle release. In contrast, membrane order, at least at the level accessible to experimental manipulation in living cells, functioned only as a modulator of the rate of release. Only a minimal trend in the apparent amount of membrane particles shed was observed across the temperature range, suggesting that there is a limit on how much is or can be released (*P* = 0.05, *r* ^2^ = 0.047 by linear regression, *n* = 5–15 per temperature, 83 total points).

It seems likely that the contribution of membrane order relates to the elasticity of the membrane. Presumably, a decrement in elasticity as temperature is raised creates flexibility in the membrane allowing for deformation sufficient for microparticles to bud and be released [41, 42]. The apparent upper boundary to the amount of particles discharged may be determined by the magnitude of the osmotically induced volume change and/or the extent of cytoskeletal alteration. The limit would be a key for cell survival since not all events resulting in microparticle shedding are associated with cell death [3].

## 5. Conclusions

The results of this study demonstrate that the biophysical mechanisms involved in microparticle release in erythrocytes and platelets probably apply broadly to all cell types. These include potassium ion efflux (presumably with concomitant osmotic effects), loss of membrane phospholipid asymmetry, and cytoskeletal disruption. Moreover, the level of membrane lipid order appears to modulate the release process with greater rates of release occurring from a more fluid membrane. This apparent effect of membrane order suggests that conditions in which these physical properties are altered may promote enhanced or inappropriate microparticle shedding. The obvious example is during apoptosis when the cell membrane becomes disordered and more fluid prior to membrane blebbing and microparticle shedding [45]. As a second example, recent investigations of blood microparticle levels of deep-sea divers have indicated an elevation of the particles after decompressing from the dive [46]. This reduction in pressure would surely have impact on the physical properties of the cell membrane similar to the elevation of temperature in Figure 4. In addition, several reports have indicated that the plasma membrane of some tumor cells is more disordered than that of the corresponding nontransformed cells [47–50]. This raises the possibility that particles shed in greater numbers from these cells could spread signals from the tumor that may impact the pathogenesis of the disease.
